# Association Between Suboccipital Muscle Tone, Postural Balance, and Head Posture in Healthy Adults

**DOI:** 10.3390/jcm15114364

**Published:** 2026-06-04

**Authors:** Jeoungeun Jeon, Youngsook Bae

**Affiliations:** 1 Department of Physical Therapy, Graduate School of Public Health, Gachon University, Incheon 21936, Republic of Korea; 2 Department of Physical Therapy, College of Medical Science, Gachon University, 191 Hambangmoe-ro, Yeonsu-gu, Incheon 21936, Republic of Korea

**Keywords:** craniovertebral angle, muscle tone, postural balance, static and dynamic balance, suboccipital muscles

## Abstract

**Background/Objectives**: The suboccipital muscles (SOMs) are rich in muscle spindles and play a critical role in proprioceptive input and postural control. However, the relationship between SOM tone, head posture, and balance performance remains unclear. Therefore, this study aimed to investigate the association between SOM tone and postural balance, including the craniovertebral angle (CVA), static balance, and dynamic balance, in healthy adults. **Methods**: A total of 112 healthy adults participated in this study. SOM, cervical extensor muscle (CEM), and upper trapezius muscle (UTM) tones were assessed. Head posture was evaluated by measuring the CVA. Static balance was assessed through the trajectory of the center of pressure (COP), whereas dynamic balance was evaluated using gait parameters. **Results**: Participants with a higher SOM tone exhibited a significantly smaller CVA, increased COP path length and velocity, and narrower step width during walking than did those with a lower tone. The regression analysis showed that SOM tone was significantly associated with CVA (β = −0.219, *p* = 0.020), COP path length (β = 0.308, *p* = 0.001) and velocity (β = 0.296, *p* = 0.002), and step width (β = −0.242, *p* = 0.014), whereas CEM and UTM tone were not significantly associated with these variables. **Conclusions:** These findings suggest that SOM tone may be associated with postural control characteristics among healthy adults.

## 1. Introduction

Muscle tone is a fundamental sensorimotor property that helps maintain postural alignment and stability through continuous neuromuscular regulation [1]. Alterations in muscle tone may arise from prolonged static postures, repetitive loading, or changes in proprioceptive input, potentially influencing postural control mechanisms [2]. Recently, MyotonPro (Myoton AS, Tallinn, Estonia), a noninvasive device that quickly and conveniently assesses the mechanical properties of muscles by applying a brief mechanical impulse and analyzing the resulting oscillatory response, has been used to objectively quantify muscle tone [3].

The cervical spine plays a critical role in postural regulation, as it integrates proprioceptive, vestibular, and visual inputs to maintain head and body alignment [4]. Particularly, the cervical extensor muscles (CEMs) and upper trapezius muscle (UTM) contribute to segmental stability and control of anterior head alignment during daily activities [5]. However, in individuals with postural abnormalities, such as forward-head posture, the muscular endurance of the cervical extensors tends to decrease, thereby reducing functional efficiency. This can lead to neck pain and abnormal cervical alignment [6]. Among the cervical musculature, the suboccipital muscles (SOMs) have unique anatomical and functional characteristics. These muscles, including the rectus capitis posterior major and minor and the obliquus capitis superior and inferior, exhibit a particularly high density of muscle spindles, suggesting a specialized role in proprioceptive processing and fine postural adjustments [7]. Due to this anatomical specificity, the SOMs are considered critical contributors to head position sense and sensorimotor integration [8]. In addition to the SOMs, the CEMs and UTM are essential for maintaining head and neck alignment and postural stability [5]. SOMs reportedly correlate with the balance capabilities of individuals with chronic stroke [9]. Furthermore, changes in cervical muscle function substantially affect postural control [10]. For instance, cervical muscle fatigue has been associated with greater postural sway and impaired balance performance [11].

Postural control is achieved through the integration of vestibular, visual, and proprioceptive inputs with appropriate muscular responses to shifts in the center of gravity [12]. Cervical muscles play crucial roles in sensory integration and motor response processes. Fatigue in the neck muscles can distort afferent signals to the central nervous system, leading to postural instability [13], which, in turn, may impair postural control, a fundamental prerequisite for functional gait [14]. In this context, cervical muscle fatigue can reduce proprioceptive sensitivity and negatively affect both static and dynamic balance performance [15]. Among the cervical muscles, the SOMs contain a higher density of muscle spindles than that in other cervical extensors, enabling more specialized roles in movement detection and postural adjustment [16]. Therefore, cervical proprioceptive input is essential for maintaining postural stability, with deficits in this system associated with diminished balance in both healthy individuals and clinical populations [17]. Given their high proprioceptive capacity, the SOMs may play a more prominent role in sensorimotor control and balance regulation than do other cervical extensors. Therefore, increased tone or fatigue in the SOMs, CEMs, or UTM may influence the static and dynamic balance abilities, which are of notable clinical relevance. Although previous studies have reported that the SOMs, CEMs, and UTM are involved in controlling head posture [18], their specific contributions to postural alignment and balance remain unclear.

Despite the well-established importance of cervical muscles in head posture control and postural regulation, limited research has specifically examined the influence of SOM tone on postural alignment and balance performance compared with that of other cervical extensors. The problem is the lack of objective and reliable tools to evaluate deep cervical muscle tone caused by these factors. Additionally, most prior research has investigated cervical proprioception, posture, or balance as distinct constructs rather than examining their interrelationships.

Accordingly, this study aimed to investigate the associations between head and neck extensor muscle tone and postural outcomes, including craniovertebral angle (CVA), static balance, and dynamic balance, in healthy adults without neck pain. We hypothesized that increased SOM tone would be associated with a smaller CVA, increased postural sway reflected by COP variables during static balance, and altered gait characteristics during dynamic balance.

## 2. Materials and Methods

### 2.1. Participants and Procedures

This cross-sectional study recruited 112 healthy adults aged 21–35 years from a university and fitness centers, using posters and telephone interviews, and included both male and female participant.

Participants were recruited through convenience sampling from the local community and university populations. Eligibility criteria included: no history of diagnosed cervical disorders and either an absence of current neck pain or only minimal discomfort—defined by a score < 2 on the numeric rating scale. Participants were excluded if they met any of the following criteria: (1) a history of cervical spine surgery; (2) cervical spine disorders including disc herniation or fracture; (3) vestibular disorders or dizziness; (4) neurological or musculoskeletal conditions affecting postural control or balance; or (5) a body mass index (BMI) ≥ 30 kg/m^2^, given the established association between elevated BMI and impaired balance ability [19].

**Figure 1:**
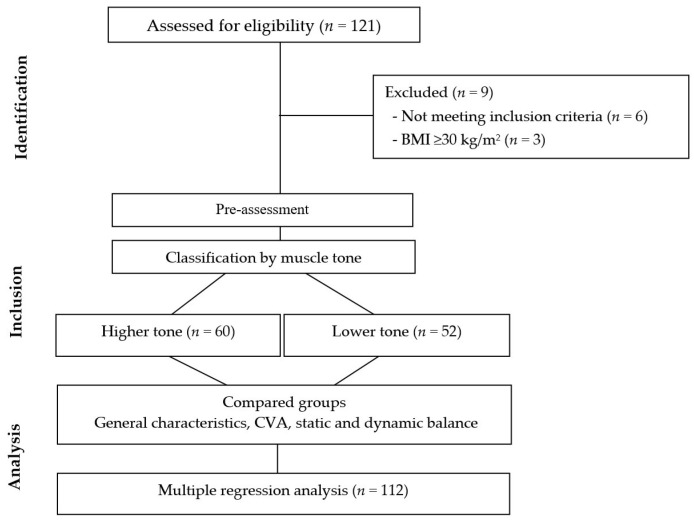
Flow diagram of this study. Abbreviations: BMI, body mass index; CVA, craniovertebral angle.

After an initial assessment of the participants’ general characteristics, the CVA was measured. Subsequently, the muscle tones of the SOMs, CEMs, and UTM were assessed. Next, the participants underwent static balance assessment, followed by an evaluation of dynamic balance during treadmill walking using gait parameters. A total of 121 individuals were screened. Of these, six were excluded because they did not meet the inclusion criteria, including individuals who reported neck pain exceeding the acceptable threshold and those with a history of cervical spine surgery or other relevant medical conditions. An additional three individuals were excluded due to a BMI ≥ 30 kg/m^2^ (Figure 1).

The study was conducted between 26 February and 30 April 2025. The sample size was determined using G*Power version 3.1.2 (Franz Faul, University of Kiel, Kiel, Germany). Sample size estimation was performed using G*Power based on the planned regression analyses examining the associations between muscle tone and postural variables. Assuming a medium effect size (f^2^ = 0.15), a significance level of 0.05, and a statistical power of 0.80, the required minimum sample size was estimated to be 92 participants according to Cohen’s criteria [20]. The required minimum sample size was estimated to be 84 participants. Considering possible dropouts and data exclusions, 121 participants were initially screened. Data were collected in a controlled laboratory environment at the university.

All assessments were performed using standardized protocols to ensure consistency and reliability of the measurements. To minimize potential bias, the evaluators were blinded to the specific objectives of the study. This blinding process was implemented to ensure the objectivity and reliability of the data collected. This study was approved by the Institutional Review Board (1044396-202501-HR-002-01; approval date: 25 February 2025). All procedures involving human participants were performed in accordance with the ethical standards of the institutional and/or national research committee and the 1964 Declaration of Helsinki and its later amendments. Written informed consent was obtained from all participants before enrollment. Participants were informed about the study’s purpose, procedures, risks, and right to withdraw at any time. Privacy and confidentiality of all participant data were maintained throughout the study.

### 2.2. Outcome Measurements

#### 2.2.1. Craniovertebral Angle

CVA measurement is considered more appropriate in the standing posture than in the sitting posture for healthy young adults [21]. Therefore, in this study, CVA was assessed by analyzing lateral photographs of participants in the standing position using digital image analysis. During the measurement, the participants were instructed to stand upright with their feet together, arms relaxed at their sides, and gaze directed forward at eye level while maintaining a static posture for a few seconds. To ensure accurate measurements, a marker was attached to the spinous process of the seventh cervical vertebra (C7) using adhesive tape, and the tragus of the ear was kept visible within the camera frame. The camera (Samsung NX Mini; Samsung Corporation, Seoul, Republic of Korea) was mounted on a horizontal tripod at shoulder height and positioned 1 m away from the participant to capture the lateral image. The CVA was defined as the angle formed between a horizontal line and a line connecting the tragus and the spinous process of C7 [22].

The captured images were analyzed using Kinovea version 0.8.15 (Kinovea, Nouvelle-Aquitaine, France). The validity (r = 0.89) and reliability (95% confidence interval = 0.78–0.99) of this photogrammetric method for CVA measurement have been previously demonstrated [23].

#### 2.2.2. Muscle Tone

**Figure 2:**
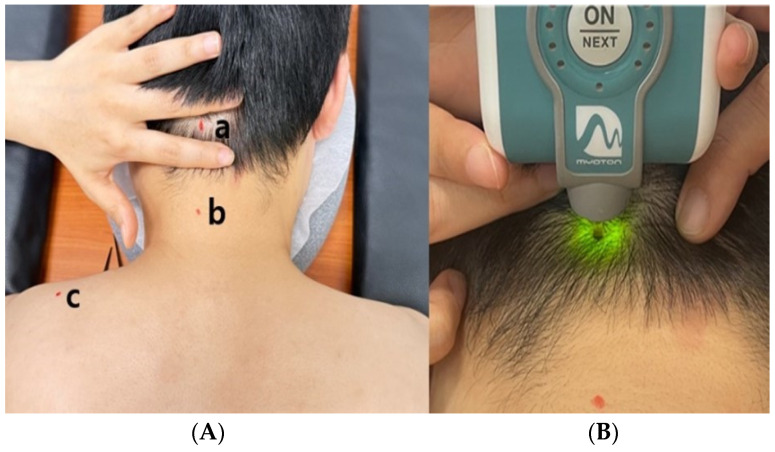
(**A**) Tone measurement points of the extensor muscles of the head and neck: (a) suboccipital muscle, (b) cervical erector spinae muscle, and (c) upper trapezius muscle. (**B**) Measurement of the suboccipital muscles using MyotonPro.

In this study, the tones of the capital and CEMs were measured using the MyotonPro device. During the measurement, the participants sat with their arms relaxed beside their thighs and leaned forward onto a treatment table in the prone position, resting their heads on a pad to maintain a comfortable posture. Muscle tone measurements were performed at standardized anatomical landmarks for each muscle. For the SOMs, the spinous process of the second cervical vertebra (C2) and the midpoint of the occipital region were palpated, and a straight line was drawn between these two landmarks using a removable skin marker [24]: 2 cm lateral to the spinous process of the fourth cervical vertebra (C4) for the semispinalis capitis, and 2 cm lateral to the midpoint between the acromion and the spinous process of C7 for the upper trapezius [25] (Figure 2A). To ensure accurate measurement of SOMs, any hair covering the measurement site was either removed or pinned to fully expose the skin before assessment (Figure 2B).

The measurements were performed in the multiscan mode with five mechanical taps set to 5 and an impulse duration (tap time) set to 15 ms. During measurement, the probe was maintained perpendicular to the skin surface at the target site. To ensure accuracy, each site was measured twice, and if the coefficient of variation exceeded 3%, the measurement was repeated. The mean of two valid measurements was used in the analysis [26]. MyotonPro provides a measure of the mechanical oscillation frequency of muscle tissue, indicating the number of oscillations per second, with units expressed in hertz (Hz), and higher values reflect greater muscle tone. MyotonPro demonstrated excellent intra-rater and inter-rater reliability, with an intraclass correlation coefficient of 0.91 [27].

#### 2.2.3. Static Balance

Static balance was assessed by measuring postural sway, defined as the displacement of the center of pressure (COP) under the eyes-open and eyes-closed conditions. COP parameters have high intrasubject reliability and can sensitively detect individual movement patterns [28]. COP displacement was recorded using a Zebris FDM-T force platform (Zebris Medical GmbH, Isny im Allgäu, Germany), which collected and analyzed data during gait and static standing trials. The Zebris FDM-T consists of an electronic mat embedded with 10,240 pressure sensors, each measuring approximately 0.85 × 0.85 cm, and collects data at a sampling rate of 120 Hz. The dedicated software automatically calculates the COP parameters from the raw data collected by the platform.

The primary outcome variables in this study included COP path length (cm), COP speed (cm/s), and directional deviations in the mediolateral and anteroposterior axes. The participants were instructed to assume a static position with their arms comfortably at their sides and feet together, with their eyes open and closed for 60 s. COP data from the 10- to 50 s interval (40 s) were extracted for analysis. The order of the eyes open and closed conditions was randomized to minimize order effects, and the first testing condition was determined through random allocation before assessment. There was a break of approximately 1 min between the two conditions. Each condition was measured three times with a 10 s break between measurements, and the average value was used.

#### 2.2.4. Dynamic Balance

Dynamic balance was assessed using spatiotemporal gait parameters measured using a Zebris FDM-T treadmill system. This device provides objective numerical values for spatiotemporal parameters during walking, enabling the quantitative analysis of gait characteristics. Before data collection, participants underwent a familiarization period on the treadmill for approximately 1–2 min to minimize the potential influence of treadmill adaptation and ensure stable gait performance. Prior to measurement, the participants were instructed to remove their shoes and walk 4 m to determine their comfortable walking speed. Based on this speed, they walked on the treadmill at their preferred pace for 80 s. To enhance data accuracy, only the middle 60 s of the data, excluding the initial and final 10 s, were used for analysis. Measurements were performed thrice, and the average of the three measurements was used. There was a rest period of approximately 1 min between measurements.

The following spatiotemporal gait parameters were measured: stance phase, single support, double stance, step length, stride length, step width, velocity, cadence, heel pressure, midfoot pressure, and toe pressure. The stance phase was defined as the period from heel-strike to toe-off. Single support refers to the time (%) spent bearing weight on one foot, and double stance indicates the time (%) when both feet are in contact with the ground. Gait velocity was calculated as the distance traveled over time (km/h), and cadence was defined as the number of steps per minute (steps/min).

The non-dominant side may demonstrate increased mediolateral and anteroposterior sway compared with that of the dominant side, which could contribute to variations in postural control [29,30]. Furthermore, normal gait is not entirely symmetrical, with limb dominance potentially affecting mediolateral force production and postural control characteristics during ambulation [31]. Therefore, the dominant side was consistently selected for analysis to reduce variability associated with limb asymmetry and maintain measurement consistency across participants. Accordingly, in this study, SOM, CEM, and UTM tone and gait assessments were performed exclusively on the dominant side to maintain consistency and standardization of measurements among participants.

Dominance was determined based on the leg used to kick the ball [32]. The independent variables in this study were CVA, static balance, and dynamic balance. The predictor variables were the tone of SOMs, CEMs, and UTM. Age and BMI were considered potential confounding variables.

### 2.3. Statistical Analyses

Statistical analyses were performed using SPSS Statistics version 22.0 (IBM Corp., Armonk, NY, USA). The normality of the data was assessed using the Shapiro–Wilk test. Descriptive statistics were used to present the participants’ general characteristics. Categorical variables were analyzed using frequency analysis, and continuous variables were expressed as mean ± standard deviation.

Participants were classified as having high muscle tone (HT) or low muscle tone (LT) based on the muscle tone of the head and neck extensor muscles, using K-means cluster analysis. K-means clustering is a widely used partitioning technique for delineating homogeneous subgroups by minimizing intra-cluster variance and maximizing inter-cluster separation [33]. In clinical research, clustering approaches are used to identify subgroups or phenotypes with shared characteristics, thereby facilitating the analysis and interpretation of complex datasets [34]. In the present study, K-means clustering with k = 2 was applied to classify participants into HT and LT groups according to muscle tone values. Two clusters were selected to facilitate clinically interpretable comparisons between individuals with relatively higher and lower muscle tone characteristics. Because categorization of continuous variables may result in information loss and reduced statistical power, HT/LT grouping was used primarily for comparative purpose. Differences between groups were evaluated using multivariate analysis of variance (MANOVA) to account for multiple dependent variables (COP measures and gait parameters) and control for potential Type I error. To mitigate potential bias arising from dichotomization and to preserve the continuous nature of the data, multiple regression analyses were additionally conducted using muscle tone as a continuous variable. Multiple regression analysis was performed to examine the effects of SOM, CEM, and UTM tones on CVA and static and dynamic balance variables, with age, sex, and BMI adjusted as covariates. Statistical significance was set at α = 0.05 for all analyses.

## 3. Results

The participants in this study were divided into HT (*n* = 60) and LT (*n* = 52) groups, and there were significant differences in CVA, SOM, and CEM tone between the two groups (Table 1).

For static balance, there were significant differences in COP path length (*p* = 0.031, η^2^ = 0.043) and velocity (*p* = 0.020, η^2^ = 0.049) under the eyes-open condition. For dynamic balance, step width significantly differed between the two groups (*p* = 0.012, η^2^ = 0.056) (Table 2 and Table 3).

When age and BMI were included as covariates, the effects of SOMs, CEMs, and UTM on CVA and the variables that differed between the two groups were analyzed. The results showed that SOMs significantly influenced CVA (*p* = 0.020, β = −0.219), COP path length (*p* = 0.001, β = 0.308) and velocity (*p* = 0.002, β = 0.296), and step width (*p* = 0.014, β = −0.242). Specifically, a higher SOM tone was associated with lower CVA values, which may indicate a tendency toward a more forward head posture. Higher SOM tone was also significantly associated with greater COP path length and velocity, as well as a narrower step width (Table 4).

## 4. Discussion

This study investigated the relationships between the muscle tones of the SOMs, CEMs, and UTM and head posture, as well as static and dynamic balance, in healthy adults. The results revealed that individuals with a higher tone in the cervical extensors showed a significantly reduced CVA and exhibited significant differences in both static and dynamic balance. In particular, an increased SOM tone was significantly associated with both static and dynamic balance variables. These findings suggest that excessive SOM tension may be associated with alterations in proprioceptive feedback in the upper cervical region, which may potentially relate to changes in head posture and balance.

Fatigue or excessive muscle tone caused by repetitive use may diminish proprioceptive acuity, thereby reducing the accuracy of joint position sense [35]. Muscle spindles play a crucial role in regulating movement and posture, and the SOMs contain more muscle spindles than those in any other muscle in the upper cervical region [36]. Because of this characteristic, the SOMs are regarded as significant contributors in supplying rich proprioceptive input and enabling precise control of head position. In the present study, participants with a higher tone in the head and neck extensors exhibited a relatively decreased CVA. In particular, the tone of the SOMs was significantly associated with lower CVA value, whereas CEM and UTM tones were not significantly associated with CVA. According to previous studies, these muscles tend to exhibit excessive activation when the CVA decreases to approximately 50° or lower [37]. Given that the mean CVA of the HT group in this study was approximately 53°, the head posture was likely insufficient to induce excessive activation of the CEMs and UTM. This observation suggests that increased muscle tone in the suboccipital region may be associated with suboptimal head and neck alignment. This association may also correspond to differences in head-position sense and subtle postural characteristics.

Previous studies have also reported that a reduction in the head angle may impair proprioception [35], thereby negatively affecting postural control, and have emphasized the importance of cervical neuromuscular function in maintaining postural balance [38]. Furthermore, Kang et al. [39] found that decreased CVA in computer workers was associated with postural imbalance. Similarly, in healthy adults, increased neck muscle tone is associated with decreased static and dynamic balance performance [40]. In the eyes-open condition of the current study, a higher SOM tone was associated with increased COP path length and velocity compared with CEM and UTM tones. These findings suggest that SOM tone may be associated with specific postural control characteristics under eyes-open conditions. Postural control relies on the integration of various sensory inputs, including visual, vestibular, and proprioceptive information, and is achieved through coordinated motor responses to COP displacement [41,42]. When visual input is unavailable, somatosensory feedback from the soles, proprioceptive information from the lower extremities, and the vestibular system may compensate to preserve postural stability [41]. The SOMs are integral to head position sense and eye–head coordination owing to their high density of muscle spindles [18]. Cervical sensorimotor control encompasses the integration of visual, vestibular, and cervical proprioceptive inputs [43]. Changes in cervical proprioception processing and sensorimotor integration strategies under eyes-closed conditions may alter cervical muscle activation patterns [44]. Therefore, these mechanisms may partially explain the more pronounced between-group differences observed under eyes-open conditions than under eyes-closed conditions in the present study. However, this interpretation remains speculative because the present study did not directly assess sensory integration or cervical proprioceptive processing mechanisms. As the participants were healthy adults, postural stability under eyes-closed conditions may have been maintained through compensatory mechanisms of sensory integration.

In this study, participants with a high SOM tone exhibited narrower step widths. A narrower step width may be related to reduced mediolateral stability characteristics because it decreases the margin of stability [45]. Step width is widely regarded as a potential indicator of mediolateral balance control in gait and is closely associated with postural stability [46]. Moreover, relaxation techniques targeting the SOMs enhance hamstring extensibility and walking speed [47,48], suggesting a link among head and neck stability, lower-limb muscle activation, and gait function. A narrower step width may indicate reduced mediolateral stability in some situations; however, it may also represent an adaptive gait strategy aimed at maintaining efficient locomotion under specific conditions. Despite the absence of significant differences in other gait parameters, the observed reduction in step width among participants with a higher SOM tone should be interpreted cautiously, as it may reflect changes in balance characteristics, adaptive strategies, or a combination of both, rather than indicating impaired balance alone.

As demonstrated in this study, SOM tone was significantly associated with CVA and selected balance-related parameters, suggesting that the SOMs may be more closely related to specific aspects of postural control than CEM or UTM tone. Increased SOM tone was associated with greater anterior–posterior and mediolateral sway under the eyes-open condition and with a narrower step width during walking. This suggests changes in postural control strategies, rather than generalized balance impairment. Therefore, SOM tone may be one of several factors influencing postural control and gait-related balance.

From a clinical perspective, this study indicates that SOM tone correlates with postural alignment, balance, and gait. These findings may offer preliminary insights into the relationships among SOM tone, head posture, and postural control in rehabilitation settings.

Despite these findings, this study has some limitations. First, the participants were limited to healthy adults, which restricts the generalizability of the findings to populations with impaired balance, such as older adults or individuals with neurological conditions. Second, muscle tone was assessed only through the mechanical properties measured by MyotonPro, without additional physiological measures, such as electromyography, to capture neuromuscular activation or control mechanisms. Third, the inability to assess potential confounding factors, such as physical activity level, habitual posture, and neck discomfort, may have influenced the observed associations between muscle tone and balance performance. Future research should consider controlling for or incorporating these variables into the analyses. Fourth, Participants were classified into high- and low-tone groups using a clustering approach based on continuous muscle tone values. Although this approach facilitated clinically interpretable group comparisons, the categorization of continuous data may not have fully reflected the relationships among variables. Fifth, balance assessments were conducted under relatively short and specific conditions. Moreover, given that this study is cross-sectional, causal relationships cannot be inferred; therefore, longitudinal studies are warranted to elucidate the direction of the relationship between muscle tone and functional outcomes. Additionally, future studies involving clinical populations with abnormal muscle tone will further improve clinical relevance. Expanding future studies to encompass broader demographic groups, incorporating comprehensive assessments of both static and dynamic balance across various conditions, and investigating the potential effects of interventions targeting SOM tone on postural control and gait-related balance characteristics will enhance the robustness and applicability of these findings.

## 5. Conclusions

This study investigated the relationship between SOM tone, CVA, and static and dynamic balance-related parameters in healthy adults. The results showed that increased SOM tone was associated with changes in head posture, greater postural sway under eyes-open conditions, and narrower step widths during walking. These findings suggest that SOM tone may be more strongly associated with specific aspects of postural control than CEM or UTM tone. Therefore, SOM tone may have clinical relevance for assessing postural control and gait-related balance characteristics.

## Figures and Tables

**Table 1:** General characteristics of the participants.

	High Muscle Tone (*n* = 60)	Low Muscle Tone (*n* = 52)	t/X^2^	*p*
Sex (Male)	33 (55.0%)^a^	33 (63.5%) ^a^	0.824	0.442
Age (Years)	25.86 ± 3.97	24.67 ± 3.41	1.691	0.094
Weight (kg)	67.99 ± 8.51	70.47 ± 11.30	1.323	0.189
Height (cm)	170.26 ± 7.22	169.97 ± 8.49	0.199	0.843
Dominant leg (Right)	30 (50.0%) ^a^	28 (53.8%) ^a^	0.165	0.708
BMI (kg/cm^2^)	23.43 ± 2.35	24.30 ± 2.89	1.754	0.082
CVA (°)	53.81 ± 6.20	55.89 ± 4.40	2.011	0.047
SOM tone (Hz)	24.37 ± 2.05	19.54 ± 1.57	13.779	<0.001
CEM tone (Hz)	16.70 ± 1.92	15.53 ± 1.48	3.557	0.001
UTM tone (Hz)	16.76 ± 1.66	16.35 ± 1.46	1.389	0.168

^a^ a number (percentage), Values are presented as mean ± standard deviation. Abbreviations: BMI, body mass index; CVA, craniovertebral angle; SOM, suboccipital muscles; CEM, cervical erector muscles; UTM, upper trapezius muscle.

**Table 2:** Comparison of static balance variables between high and low muscle tones.

	High Muscle Tone	Low Muscle Tone	F	*p*	η^2^
Eye open					
COP path length (mm)	288.28 ± 122.98	239.86 ± 103.95	4.799	0.031	0.043
COP velocity (mm/s)	8.68 ± 3.94	3.94 ± 2.37	5.592	0.020	0.049
ML deviation(mm)	6.82 ± 3.62	7.21 ± 4.52	0.823	0.366	0.008
AP deviation (mm)	11.37 ± 5.31	9.52 ± 4.92	0.888	0.348	0.008
Eye closed					
COP path length (mm)	348.60 ± 142.84	311.79 ± 157.76	0.759	0.386	0.007
COP velocity (mm/s)	11.37 ± 5.31	9.52 ± 4.92	2.196	0.141	0.020
ML deviation (mm)	6.48 ± 3.60	7.34 ± 4.25	1.669	0.199	0.015
AP deviation (mm)	20.25 ± 11.37	20.84 ± 11.51	0.151	0.698	0.001

Abbreviations: COP, center of pressure; ML, mediolateral deviation; AP, anteroposterior.

**Table 3:** Comparison of dynamic balance variables between high and low muscle tones.

	High Muscle Tone	Low Muscle Tone	F	*p*	η^2^
Stance phase (%)	63.66 ± 1.71	63.56 ± 1.70	0.100	0.752	0.001
Single support (%)	36.25 ± 1.73	35.97 ± 1.41	0.892	0.347	0.008
Double stance (%)	27.41 ± 3.22	27.61 ± 2.95	0.106	0.745	0.001
Stride length (cm)	112.09 ± 11.01	111.56 ± 11.51	0.063	0.802	0.001
Step width (cm)	9.60 ± 2.38	10.76 ± 2.37	6.575	0.012	0.056
Velocity (km/h)	3.75 ± 0.91	3.78 ± 1.04	0.022	0.881	0.000
cadence (Step/min)	106.19 ± 12.68	106.96 ± 15.08	0.086	0.770	0.001

**Table 4:** Multivariate regression analysis to identify factors affecting head posture and static and dynamic balance.

	CVA (β/*p*)	COP Path Length (β/*p*)	COP Velocity (β/*p*)	Step Width (β/*p*)
SOM tone	−0.219/0.020	0.308/0.001	0.296/0.002	−0.242/0.014
CEM tone	−0.099/0.341	0.100/0.287	0.103/0.269	0.050/0.606
UTM tone	0.057/0.543	0.156/0.087	0.196/0.060	0.015/0.876
Adj.R^2^	0.033	0.112	0.120	0.029

Statistical analyses were performed by adjusting for age and body mass index as covariates. Abbreviations: SOM, suboccipital muscle; CEM, cervical erector muscle; UTM, upper trapezius; CVA, craniovertebral angle; COP, center of pressure.

## Data Availability

The data presented in this study are available on request from the corresponding author due to participant privacy concerns.

## Abbreviations

The following abbreviations are used in this manuscript:

SOMs	Suboccipital muscles
CEMs	Cervical extensor muscles
UTM	Upper trapezius muscle
CVA	Craniovertebral angle
COP	Center of pressure
HT	High muscle tone group
LT	Low muscle tone group
BMI	Body mass index

## References

[1] Bhimani R.H., Gaugler J.E., Skay C. (2017). Understanding symptom experiences of muscle tightness from patients’ and clinicians’ perspectives. J. Clin. Nurs..

[2] Gosain L., Ahmad I., Rizvi M.R., Sharma A., Saxena S. (2022). Prevalence of musculoskeletal pain among computer users working from home during the COVID-19 pandemic: A cross-sectional survey. Bull. Fac. Phys. Ther..

[3] Agyapong-Badu S., Warner M., Samuel D., Stokes M. (2016). Measurement of ageing effects on muscle tone and mechanical properties of rectus femoris and biceps brachii in healthy males and females using a novel hand-held myometric device. Arch. Gerontol. Geriatr..

[4] Boyd-Clark L., Briggs C., Galea M. (2002). Muscle spindle distribution, morphology, and density in longus colli and multifidus muscles of the cervical spine. Spine.

[5] Cheragh Z.A., Gandomi F., Sakinehpoor A. (2023). Effects of typing positions on the upper trapezius and neck extensor muscles electromyography in office employees: A single-blind cross-sectional study. Work.

[6] Lotfian S., Fesharaki M.J., Shahabbaspour Z., Akbarzadeh H., Moezy A. (2025). The impact of forward head posture on neck muscle endurance and thickness in women with chronic neck pain: A cross-sectional study. BMC Musculoskelet. Disord..

[7] La Rocca G., Altieri R., Ricciardi L., Olivi A., Della Pepa G.M. (2017). Anatomical study of occipital triangles: The ‘inferior’suboccipital triangle, a useful vertebral artery landmark for safe postero-lateral skull base surgery. Acta Neurochir..

[8] Pettorossi V.E., Schieppati M. (2014). Neck proprioception shapes body orientation and perception of motion. Front. Hum. Neurosci..

[9] Nam M.-J., Kim J.-H., Shin Y.-J., Kim M.-K. (2025). Effects of Suboccipital Muscle Release on Balance and Gait in Patients with Chronic Stroke: A Case Study. Korean Soc. Phys. Med..

[10] Kristjansson E., Treleaven J. (2009). Sensorimotor function and dizziness in neck pain: Implications for assessment and management. J. Orthop. Sports Phys. Ther..

[11] Gosselin G., Fagan M.J. (2014). The effects of cervical muscle fatigue on balance—A study with elite amateur rugby league players. J. Sports Sci. Med..

[12] Pollock A.S., Durward B.R., Rowe P.J., Paul J.P. (2000). What is balance?. Clin. Rehabil..

[13] Schieppati M., Nardone A., Schmid M. (2003). Neck muscle fatigue affects postural control in man. Neuroscience.

[14] Patikas D. (2015). Gait and balance. Comorbid Conditions in Individuals with Intellectual Disabilities.

[15] Abdelkader N.A., Mahmoud A.Y., Fayaz N.A., Mahmoud L.S.E.-D. (2020). Decreased neck proprioception and postural stability after induced cervical flexor muscles fatigue. J. Musculoskelet. Neuronal Interact..

[16] Liu J.-X., Thornell L.-E., Pedrosa-Domellöf F. (2003). Muscle spindles in the deep muscles of the human neck: A morphological and immunocytochemical study. J. Histochem. Cytochem..

[17] Liang Z., Clark R., Bryant A., Quek J., Pua Y.H. (2014). Neck musculature fatigue affects specific frequency bands of postural dynamics during quiet standing. Gait Posture.

[18] Sung Y.-H. (2022). Suboccipital muscles, forward head posture, and cervicogenic dizziness. Medicina.

[19] Mocanu G.D., Murariu G. (2022). The association of gender and body mass index on the values of static and dynamic balance of university students (a cross-sectional design study). Appl. Sci..

[20] Cohen J. (2013). Statistical Power Analysis for the Behavioral Sciences.

[21] Titcomb D.A., Melton B.F., Bland H.W., Miyashita T. (2024). Evaluation of the Craniovertebral Angle in Standing versus Sitting Positions in Young Adults with and without Severe Forward Head Posture. Int. J. Exerc. Sci..

[22] Singla D., Veqar Z., Hussain M.E. (2017). Photogrammetric assessment of upper body posture using postural angles: A literature review. J. Chiropr. Med..

[23] Li C., Zhao Y., Yu Z., Han X., Lin X., Wen L. (2022). Sagittal imbalance of the spine is associated with poor sitting posture among primary and secondary school students in China: A cross-sectional study. BMC Musculoskelet. Disord..

[24] Lee H.-j., Lee Y.-s., Jeong J.-y., Seo D.-k. (2019). Correlation between tone of suboccipital muscle and endurance of deep neck flexor muscle according to angle changes in college students. J. Korean Soc. Phys. Med..

[25] Taş S., Yaşar Ü., Kaynak B.A. (2021). Interrater and intrarater reliability of a handheld myotonometer in measuring mechanical properties of the neck and orofacial muscles. J. Manip. Physiol. Ther..

[26] Lettner J., Królikowska A., Ramadanov N., Oleksy Ł., Hakam H.T., Becker R., Prill R. (2024). Evaluating the reliability of MyotonPro in assessing muscle properties: A systematic review of diagnostic test accuracy. Medicina.

[27] Valenti F., Meden S., Frangež M., Vauhnik R. (2024). Intra-rater and inter-rater reliability of a handheld myotonometer measuring myofascial stiffness of lower lumbar myofascial tissue in healthy adults. PeerJ.

[28] Li Z., Liang Y.-Y., Wang L., Sheng J., Ma S.-J. (2016). Reliability and validity of center of pressure measures for balance assessment in older adults. J. Phys. Ther. Sci..

[29] Sung P.S., Lee D. (2025). Comparative analysis of dominant limb postural control in adults with and without fear of falling. BMC Geriatr..

[30] Promsri A., Haid T., Werner I., Federolf P. (2020). Leg dominance effects on postural control when performing challenging balance exercises. Brain Sci..

[31] Polk J.D., Stumpf R.M., Rosengren K.S. (2017). Limb dominance, foot orientation and functional asymmetry during walking gait. Gait Posture.

[32] van Melick N., Meddeler B.M., Hoogeboom T.J., Nijhuis-van der Sanden M.W., van Cingel R.E. (2017). How to determine leg dominance: The agreement between self-reported and observed performance in healthy adults. PLoS ONE.

[33] Taroi M., Gligorea I., Fleacă R., Vecerzan L., Prihoi A., Domnariu C.-D. (2025). Profiling Patients with Chronic Ulcers Using K-Means Clustering and Analysis of the Impact on the Consumption of Medical Resources: Retrospective Study on Hospitalized Patients in Romania. J. Clin. Med..

[34] Nalinthasnai N., Thammasudjarit R., Tassaneyasin T., Eksombatchai D., Sungkanuparph S., Boonsarngsuk V., Sutherasan Y., Junhasavasdikul D., Theerawit P., Petnak T. (2025). Unsupervised machine learning clustering approach for hospitalized COVID-19 pneumonia patients. BMC Pulm. Med..

[35] Karagiannopoulos C., Watson J., Kahan S., Lawler D. (2020). The effect of muscle fatigue on wrist joint position sense in healthy adults. J. Hand Ther..

[36] Kulkarni V., Chandy M., Babu K. (2001). Quantitative study of muscle spindles in suboccipital muscles of human foetuses. Neurol. India.

[37] Lee S., Lee D., Park J. (2015). Effect of the cervical flexion angle during smart phone use on muscle fatigue of the cervical erector spinae and upper trapezius. J. Phys. Ther. Sci..

[38] Vuillerme N., Pinsault N., Vaillant J. (2005). Postural control during quiet standing following cervical muscular fatigue: Effects of changes in sensory inputs. Neurosci. Lett..

[39] Kang J.-H., Park R.-Y., Lee S.-J., Kim J.-Y., Yoon S.-R., Jung K.-I. (2012). The effect of the forward head posture on postural balance in long time computer based worker. Ann. Rehabil. Med..

[40] Tian M.-Y., Lee M.-H., Kim M.-K. (2025). Effects of Neck Muscle Tone on Static and Dynamic Balance in Healthy Participants. J. Korean Soc. Phys. Med..

[41] Shumway-Cook A., Woollacott M.H. (2007). Motor Control: Translating Research into Clinical Practice.

[42] Ivanenko Y., Gurfinkel V.S. (2018). Human postural control. Front. Neurosci..

[43] Peng B., Yang L., Li Y., Liu T., Liu Y. (2021). Cervical proprioception impairment in neck pain-pathophysiology, clinical evaluation, and management: A narrative review. Pain Ther..

[44] Wei W., Li W., Wang Y., Zhang S., Fan G., Bai Y. (2026). Importance of visual and proprioceptive inputs for maintaining balance in patients with chronic non-specific neck pain: A cross-sectional study. PLoS ONE.

[45] Yoon J.-Y., Shin S.-S. (2024). Impact of step width on trunk motion and gait adaptation in elderly women with knee osteoarthritis. J. Back Musculoskelet. Rehabil..

[46] Molina L.K., Small G.H., Neptune R.R. (2023). The influence of step width on balance control and response strategies during perturbed walking in healthy young adults. J. Biomech..

[47] Wannaprom N., Treleaven J., Jull G., Uthaikhup S. (2018). Neck muscle vibration produces diverse responses in balance and gait speed between individuals with and without neck pain. Musculoskelet. Sci. Pract..

[48] Cho S.H., Kim S.H., Park D.J. (2015). The comparison of the immediate effects of application of the suboccipital muscle inhibition and self-myofascial release techniques in the suboccipital region on short hamstring. J. Phys. Ther. Sci..

